# Verifying the Feasibility of Implementing Semantic Interoperability in Different Countries Based on the OpenEHR Approach: Comparative Study of Acute Coronary Syndrome Registries

**DOI:** 10.2196/31288

**Published:** 2021-10-19

**Authors:** Lingtong Min, Koray Atalag, Qi Tian, Yani Chen, Xudong Lu

**Affiliations:** 1 School of Electronics and Information Northwestern Polytechnical University Xi'an China; 2 Auckland Bioengineering Institute University of Auckland Auckland New Zealand; 3 College of Biomedical Engineering & Instrument Science Zhejiang University Hangzhou China

**Keywords:** semantic interoperability, openEHR, archetype, registry, acute coronary syndrome

## Abstract

**Background:**

The semantic interoperability of health care information has been a critical challenge in medical informatics and has influenced the integration, sharing, analysis, and use of medical big data. International standard organizations have developed standards, approaches, and models to improve and implement semantic interoperability. The openEHR approach—one of the standout semantic interoperability approaches—has been implemented worldwide to improve semantic interoperability based on reused archetypes.

**Objective:**

This study aimed to verify the feasibility of implementing semantic interoperability in different countries by comparing the openEHR-based information models of 2 acute coronary syndrome (ACS) registries from China and New Zealand.

**Methods:**

A semantic archetype comparison method was proposed to determine the semantics reuse degree of reused archetypes in 2 ACS-related clinical registries from 2 countries. This method involved (1) determining the scope of reused archetypes; (2) identifying corresponding data items within corresponding archetypes; (3) comparing the semantics of corresponding data items; and (4) calculating the number of mappings in corresponding data items and analyzing results.

**Results:**

Among the related archetypes in the two ACS-related, openEHR-based clinical registries from China and New Zealand, there were 8 pairs of reusable archetypes, which included 89 pairs of corresponding data items and 120 noncorresponding data items. Of the 89 corresponding data item pairs, 87 pairs (98%) were mappable and therefore supported semantic interoperability, and 71 pairs (80%) were labeled as “direct mapping” data items. Of the 120 noncorresponding data items, 114 (95%) data items were generated via archetype evolution, and 6 (5%) data items were generated via archetype localization.

**Conclusions:**

The results of the semantic comparison between the two ACS-related clinical registries prove the feasibility of establishing the semantic interoperability of health care data from different countries based on the openEHR approach. Archetype reuse provides data on the degree to which semantic interoperability exists when using the openEHR approach. Although the openEHR community has effectively promoted archetype reuse and semantic interoperability by providing archetype modeling methods, tools, model repositories, and archetype design patterns, the uncontrolled evolution of archetypes and inconsistent localization have resulted in major challenges for achieving higher levels of semantic interoperability.

## Introduction

Due to the rapid development of information and communication technologies and their continuous application in the medical domain, massive electronic medical data and information have been generated by medical information systems, services, and devices. These vast amounts of medical data and information have the potential to improve the safety and quality of medical services, reduce the cost of such services, and promote medical research [1]. The realization of this potential needs to be supported by the effective application of advanced information and communication technologies that manage medical big data, such as big data analysis technologies and artificial intelligence technologies. However, the effective application of these technologies is premised on implementing semantic interoperability, which is an indispensable basis for the construction, sharing, analysis, and use of medical big data. The semantic interoperability of clinical data and information has been a critical challenge and hot topic in medical informatics [2].

To implement and improve semantic interoperability, international standard organizations have developed approaches for constructing standards based on multilevel medical information models. Such organizations include Health Level Seven (HL7) International [3], the International Organization for Standardization (ISO) [4], and the openEHR Foundation [5].

To promote the exchange and sharing of medical information, HL7 International has developed a series of standards based on the HL7 development framework and medical information models, including the HL7 v2 and v3 messaging standards and HL7 Clinical Document Architecture standards. These standards and models have made important contributions to the improvement of semantic interoperability, especially semantic interoperability in the electronic exchange of messages and medical documents [6,7]. The HL7 Clinical Document Architecture standards have become core standards for the interoperability of medical documents. Although HL7 v3 can improve semantic interoperability, the inconsistency and complexity of its reference information model and modeling approach have limited its implementation [8].

To solve the complexity problem, HL7 International launched the Fast Healthcare Interoperability Resources (FHIR) standard and a limited set of information models to easily implement semantic interoperability [9]. FHIR provide a set of resources for expressing the structure and semantics of the most commonly exchanged information and provide a flexible extension mechanism for defining the semantics of information that is not included in FHIR. The feasibility of using FHIR to improve the semantic interoperability of medical information systems and applications has been verified [10-13].

ISO13606 is a series of health informatics standards that facilitate electronic health record (EHR) data exchange and communication between EHR systems and data repositories [14]. ISO13606 is based on the openEHR 2-level modeling method, which involves a simplified reference model, and is regarded as a subset of the openEHR specifications [15].

The openEHR Foundation provides a comprehensive information architecture for representing the entirety of EHR content in a structured and interoperable form. This architecture includes a modeling framework that uses domain-specific languages and intuitive modeling tools. The openEHR Foundation tries to achieve sustainable semantic interoperability by using a consistent multilevel modeling method to define reusable domain concepts and their formal semantics [16].

The models within the openEHR approach consist of a reference model, archetypes, and templates. The reference model is a stable information model that defines a logical medical information architecture and includes a demographic information model, an EHR information model, the EHR Extract Information model, data types, and data structures. An archetype is a reusable information model that comprises a maximal set of content element definitions and general constraints. Templates are context-specific data set definitions that are created by combining and constraining relevant archetypes for generating forms, documents, data persistence, and messages. Archetypes and templates are defined by domain experts using a formal editorial and publication process to foster semantic interoperability.

OpenEHR-related research is gradually becoming one of the most discussed semantic interoperability–related research topics. Such research involves archetype modeling [17-23], data persistence [24-26], language design [27], model mapping [28], model retrieval [29,30], and reuse [19].

The openEHR approach has been adopted in many countries to improve the semantic interoperability of medical information systems [31], such as those in Norway [30], China [17], Portugal [32], Brazil [15,33], and Germany [34]. Moreover, the feasibility of improving semantic interoperability based on the openEHR approach has been verified in many domains, including genomics [21], clinical decision support systems [34], clinical registries [35], clinical data sets [36], and EHRs [37].

To the best of our knowledge, there has been no research on verifying the feasibility of implementing semantic interoperability in different countries based on the openEHR approach.

## Methods

### Study Design

In order to verify the feasibility and degree of implementing semantic interoperability in 2 countries based on the openEHR approach, we conducted a semantic comparison of the archetypes in 2 acute coronary syndrome (ACS)–related clinical data registries from China and New Zealand.

In this paper, the analyzed ACS-related data registry from China was the Coronary Computed Tomography Angiography (CCTA) registry [35], which is used to improve the early detection of coronary atherosclerosis as part of a national key research and development project. The CCTA registry was designed to collect clinical data from patients who have undergone CCTA examination. The analyzed ACS-related registry from New Zealand was the All New Zealand ACS Quality Improvement (ANZACS-QI) program. The ANZACS-QI registry is a cardiac registry for ACS events and cardiac procedures. The data management of these two registries was based on the openEHR approach [36].

Archetype reuse is key for establishing semantic interoperability based on the openEHR approach. Therefore, the semantic expression comparison of reused archetypes can be conducted to determine the degree to which different openEHR-based clinical systems and applications achieve semantic interoperability.

**Figure 1:**
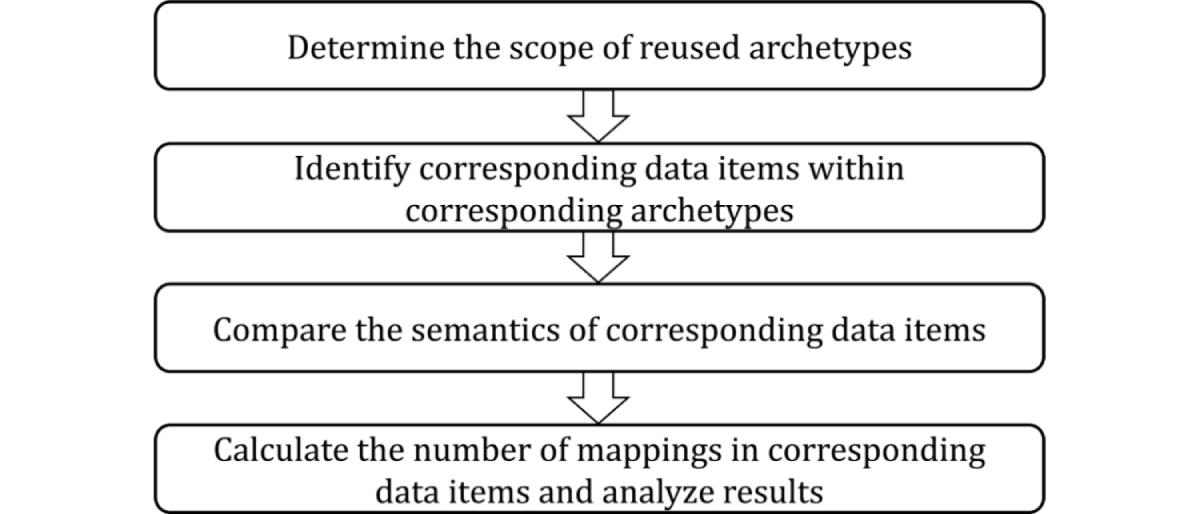
The procedure for the semantic archetype comparison method.

This paper proposes a semantic archetype comparison method for determining the degree of semantic interoperability via archetype reuse in 2 ACS-related clinical registries from 2 countries. This semantic archetype comparison method consisted of the following four steps: (1) determining the scope of reused archetypes; (2) identifying corresponding data items within corresponding archetypes; (3) comparing the semantics of corresponding data items; and (4) calculating the number of mappings in corresponding data items and analyzing results, as shown in Figure 1.

### Determining the Scope of Reused Archetypes

The scope of reused archetypes was determined based on the comparison of archetype names, types, descriptions, and metadata. An archetype name is an archetype’s unique identifier, and it reflects an archetype’s semantics, including the archetype type, corresponding domain concept, and version information. The types of archetypes include the observation, instruction, action, evaluation, and administrative archetypes.

The continuous improvement of archetypes over time results in changes in the archetype version based on certain rules. Localizing existing archetypes also leads to changes in archetype names, which usually manifest as the addition of suffixes to archetype names. Therefore, while determining the scope of reusable archetypes, if the names of 2 archetypes were the same or if there was a localization relationship between 2 archetypes, they were matched for reuse.

### Identifying Corresponding Data Items Within Corresponding Archetypes

It was necessary to identify the corresponding data items in each pair of corresponding reused archetypes. This was done by comparing the data items’ semantic descriptions. If the semantic descriptions of 2 data items were the same or similar, they were marked as corresponding data items; otherwise, they were marked as noncorresponding data items. Noncorresponding data items indicated semantic gaps or differences among the data items from reused archetypes that may hinder semantic interoperability.

### Comparing the Semantics of Corresponding Data Items

**Figure 2:**
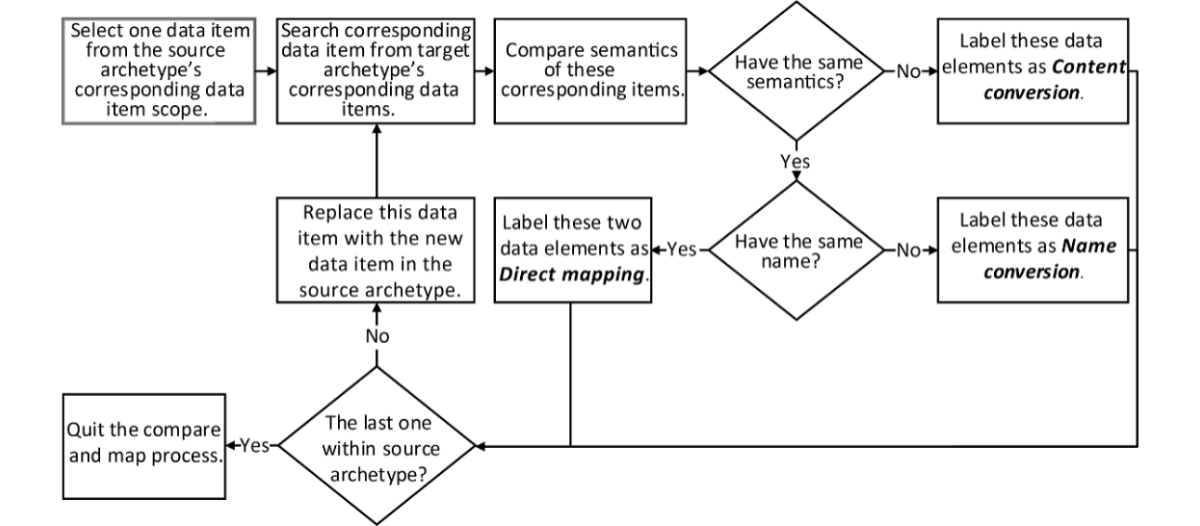
The process of the semantic archetype comparison.

The semantic comparison of the corresponding data items within reused archetypes mainly involved data element names and semantic descriptions. In accordance with this procedure, the corresponding data items within reused archetypes were labeled as “direct mapping,” “name conversion,” and “content conversion” data items based on the mapping results, as shown in Figure 2. These semantic mapping–based data item types were used to determine the degree to which an archetype’s data items supported semantic interoperability. The “direct mapping” corresponding data items could support complete semantic interoperability without human intervention. The “name conversion” corresponding data items could support semantic interoperability via manual or automated name mapping. The “content conversion” corresponding data items referred to items that made it challenging to achieve semantic interoperability.

### Calculating the Number of Mappings in Corresponding Data Items and Analyzing Results

First, the number of mappings for each data item type among the corresponding data items in each reused archetype and in all reused archetypes was calculated. Second, the direct mapping ratio and the mappable ratio of the corresponding data items in each reused archetype and in all reused archetypes were calculated. Finally, the data obtained from the abovementioned calculations were analyzed to illustrate the reusability of related archetypes from the two registries.

## Results

To illustrate the feasibility of implementing semantic interoperability between the two coronary clinical data registries based on the openEHR approach, we compared the corresponding data elements within reused archetypes by using statistical analyses.

### The Scope of Reused Archetypes and Corresponding Data Items

By comparing the ACS-related archetypes in the two registries, 8 pairs of reused archetypes were identified. These are shown in Textbox 1.

**Corresponding acute coronary syndrome–related reused archetypes from China and New Zealand.**

**Reused archetypes from China**

1. openEHR-EHR-EVALUATION.problem_diagnosis.v1
2. openEHR-EHR-ACTION.medication.v1
3. openEHR-EHR-OBSERVATION.imaging_exam_result.v0
4. openEHR-EHR-OBSERVATION.blood_pressure.v2
5. openEHR-EHR-EVALUATION.tobacco_smoking_summary.v1
6. openEHR-EHR-EVALUATION.family_history.v2
7. openEHR-EHR-OBSERVATION.body_weight.v2
8. openEHR-EHR-OBSERVATION.height.v2

**Reused archetypes from New Zealand that corresponded with those from China**

1. openEHR-EHR-EVALUATION.problem_diagnosis_nehta.v1
2. openEHR-EHR-ACTION.medication.v1
3. openEHR-EHR-OBSERVATION.imaging_exam.v1
4. openEHR-EHR-OBSERVATION.blood_pressure.v1
5. openEHR-EHR-EVALUATION.tobacco_use_summary.v1
6. openEHR-EHR-EVALUATION.family_history.v1
7. openEHR-EHR-OBSERVATION.body_weight.v1
8. openEHR-EHR-OBSERVATION.height.v1

The semantic comparison of data items in the reused archetypes revealed 89 pairs of corresponding data items and 120 noncorresponding data items from the two countries. Of the 120 noncorresponding data items, 86 were from the reused archetypes in China, and 34 were from the reused archetypes in New Zealand. Further, 50.9% (89/175) of the data items from China were corresponding data items, and 49.1% (86/175) of the data items were noncorresponding data items. Additionally, 72.4% (89/123) of the data items from New Zealand were corresponding data items, and 27.6% (34/123) of the data items were noncorresponding data items.

The reasons for and the proportion distribution of the noncorresponding data items in the reused archetypes were compared and analyzed. Of the 86 noncorresponding items from China, 83 were generated via archetype evolution and 3 were generated via archetype localization. Further, 31 of the 34 noncorresponding data items from New Zealand were generated via archetype version evolution and 3 were generated via archetype localization.

### Results of the Semantic Comparison of Corresponding Data Items

The results of the semantic comparison of corresponding data items in reused archetypes are shown in the Table 1.

**Table 1:** The results of the semantic comparison of corresponding data items in reused archetypes.

Domain concept	Total number of data items	Direct mapping data items, n	Name conversion data items, n	Content conversion data items, n	Direct mapping ratio, %	Mappable ratio, %
Diagnosis	9	3	5	1	33	89
Imaging exam	26	21	5	0	81	100
Medication	4	3	1	0	75	100
Blood pressure	16	16	0	0	100	100
Smoking history	7	6	0	1	14	86
Family history	17	12	5	0	71	100
Weight	5	5	0	0	100	100
Height	5	5	0	0	100	100
All concepts	89	71	16	2	80	98

“Direct mapping” data items referred to data items in reused archetypes that could support semantic interoperability without any modification. The semantics of the “name conversion” data items were the same, but the names of the data items were different (eg, “Problem/Diagnosis” vs “Problem/Diagnosis name” for the corresponding reused “diagnosis” archetypes). “Name conversion” data items referred to corresponding data items that could also support semantic interoperability, but some additional conversion mapping would be required. However, “content conversion” data items had the problem of incomplete semantic matching between data items and therefore limited the realization of semantic interoperability. As such, the “mappable ratio” of data items that supported semantic interoperability included the “direct mapping” and “name conversion” data items.

**Figure 3:**
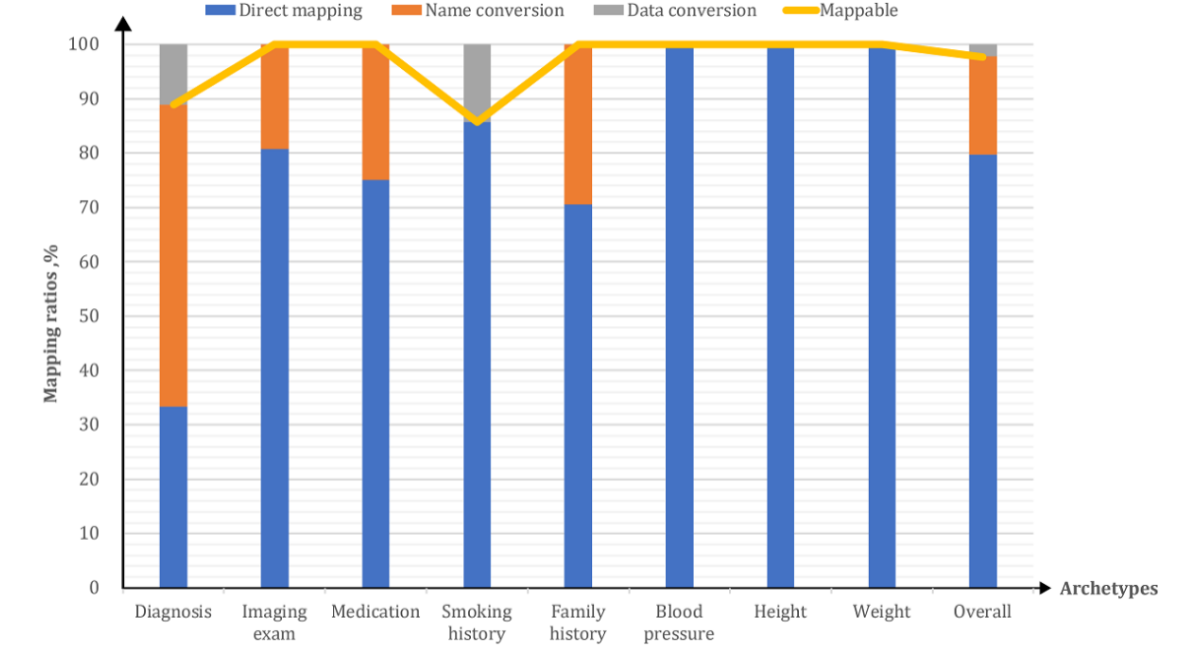
The various mapping ratios of the corresponding data items of reused archetypes.

The mapping ratios of the corresponding data items of the reused archetypes are shown in Figure 3. The mappable proportion of all corresponding data items within reused archetypes was as high as 98% (87/89). Except for the mapping ratios of the “diagnosis” (8/9, 89%) and “smoking history” (6/7, 86%) archetypes, the mapping ratios of the corresponding items of all archetypes were 100%.

## Discussion

### Principal Findings

Archetype reuse is the main determinant of the semantic interoperability of openEHR-based medical information. This study attempted to verify the feasibility of achieving semantic interoperability and the extent to which the semantic interoperability of clinical information can be achieved by assessing archetype reuse in 2 ACS registries from China and New Zealand. The results of our novel semantic comparison method indicated that the ratio of direct mappings between the corresponding data items of reused archetypes reached 98% (87/89), and of the 8 reused archetypes, 6 (75%) had data items that achieved a direct mapping ratio of 100%. This very high degree of reused archetype direct mapping proves the feasibility of achieving semantic interoperability by using the openEHR approach.

Although the two clinical data registries for China and New Zealand both contained data about ACS, they have different scopes due to their different purposes. The ANZACS-QI registry is used to support clinical quality improvement and research, whereas the CCTA registry is used to improve the early detection of coronary atherosclerosis. Therefore, the aspects that are important for New Zealand may not be as relevant to China and vice versa. For example, invasive management and emergency management are two essential parts of ACS management in New Zealand; however, the CCTA registry barely covers these aspects. Conversely, CCTA examination is the cornerstone aspect of the CCTA registry; however, this is not relevant to the ANZACS-QI registry.

For the past 2 decades, extensive research on achieving semantic interoperability through archetype reuse has been undertaken by the openEHR community. Further, valuable insights have been gained through the evaluation of real-world deployments. Although the flexibility and extensibility of the openEHR approach allow for a high degree of freedom when creating and modifying archetypes for specific purposes, it is critically important to ensure that these archetypes can be reused. To this end, extensive efforts and resources have been devoted to the development and sharing of archetypes at an international level. The development of volunteer-created editorial processes and the Clinical Knowledge Manager (CKM) model repository [38], which is freely available to the global openEHR community, were significant steps toward creating more than 500 archetypes that represent a substantial portion of EHRs. These efforts have provided an essential foundation for archetype reuse among various applications worldwide.

Although these efforts have drawn remarkable attention and have resulted in significant breakthroughs, there are still important challenges to archetype reuse among various systems and applications [5,17,19,22,23]. These challenges include (1) the uncontrolled evolution of existing archetypes across various versions of the CKM; (2) inconsistencies in new archetype development; (3) unanticipated semantic changes generated in successive versions of an archetype; and (4) inconsistencies in localization and terminology bindings.

We observed several of these issues in our study. For example, the semantic mismatch between the “openEHR-EHR-EVALUATION.problem_diagnosis.v1” archetype in the international CKM and the “openEHR-EHR-EVALUATION.problem_diagnosis_nehta.v1” archetype in the Australian National E-Health Transition Authority version of the CKM resulted in the inability to directly map all data elements. Another example is the use of two different versions of archetypes—the “openEHR-EHR-EVALUATION.family_history.v2” archetype and the “openEHR-EHR-EVALUATION.family_history.v1” archetype in the two modeling registries.

From a methodological point of view, a graphical archetype discovery method [29] and formal archetype modeling methods [17,22,23,35,39] for facilitating robust archetype development have previously been proposed and validated. Although these efforts could improve archetype reuse, achieving real-world semantic interoperability, as illustrated in this study, between 2 ACS registries largely depends on human factors. Therefore, a prescriptive modeling framework is needed. Integrating semantic-based model discovery and automated modeling assistance services into archetype development tools is a potential solution for meeting this need. However, to the best of our knowledge, such methodologies and tools do not exist and warrant future research. In addition, the establishment of archetype update notification services, which would notify developers of information systems (ie, those that use existing archetypes) about changes, can be very helpful for ensuring the continuity of semantic interoperability over time as new changes are incorporated.

This *Discussion* section would be incomplete if we did not highlight the parallels between openEHR archetypes and HL7 FHIR, as both are formal models of health care information. The comparison of the FHIR and openEHR approaches is shown in the Table 2. On one hand, while anyone can create new archetypes that meet specific requirements, FHIR do not allow for the creation of new resources but provide extensions and profiling mechanisms for customizing existing resources. On the other hand, the openEHR multilevel modeling method, which is based on a stable reference model, provides a well-defined framework for expressing complex concepts and adapts to the rapid evolution of domain concepts better than the FHIR approach. Although the centrally developed and published FHIR are concrete and immutable (ie, factors that foster model reuse), the unbounded extension mechanism still relies on human factors for aligning resources with existing extensions. Ultimately, the compromise between the freedom of creating new information models and the need to ensure semantic alignment at a global scale applies equally to both the openEHR and FHIR formalisms. Therefore, our methodology and results should also be applicable to FHIR.

**Table 2:** The comparison between the Fast Healthcare Interoperability Resources (FHIR) and openEHR approaches.

Comparison aspects	FHIR approach	OpenEHR approach
Scope and purpose	FHIR is a new Health Level Seven specification for defining the structure and semantics of health care information that is involved in health information exchange. It is not engineered for persistence and the modeling of all electronic health record data.	OpenEHR defines the structure and semantics of all health information in electronic health records and is engineered for persistence and health information exchange.
Reference model	The model does not have a separate layer and uses a mix of data types and structural resources.	The model has a discrete and stable layer that comprises building blocks, upon which archetypes are built.
Information model	FHIR	OpenEHR archetypes
Composition and constraints	FHIR profile	OpenEHR template
Extensibility	FHIR extensions that are discoverable via Uniform Resource Identifiers	New archetypes or archetype specializations for adding new data items, value sets, and tighter constraints
Localization	Localization is not well defined. Localization is possible with extensions, but this is under review.	Localization is a well-defined section in archetypes for data elements and value sets.
Terminology support	Supports terminology bindings	Supports terminology bindings
Reference relationship	The resources may refer to those outside of FHIR.	The archetypes allow for the linking of other archetypes via an archetype slotting mechanism.

Due to the ever-increasing amounts of clinical domain knowledge and various local implementation needs, the consistency and reusability of new or modified archetypes are grand challenges to implementing semantic interoperability. In our study, we were fortunate, as the number of these inconsistencies were minimal. As such, we were able to demonstrate the feasibility of achieving a very high level of semantic interoperability between 2 clinical registries from 2 very distinct countries.

### Conclusions

The feasibility of implementing semantic interoperability in 2 ACS registries that are based on the openEHR approach was verified by the results of our semantic comparison of reused archetypes. Continuous improvement and localized archetype modification may reduce the proportion of direct mappings among data items in reused archetypes and result in a gap between actual semantic interoperability and theoretical semantic interoperability. Although the openEHR community has provided an essential foundation for archetype reuse through robust editorial processes and a freely available CKM, there are still important challenges to archetype reuse.

## Abbreviations

ACS: acute coronary syndrome
ANZACS-QI: All New Zealand Acute Coronary Syndrome Quality Improvement
CCTA: Coronary Computed Tomography Angiography
CKM: Clinical Knowledge Manager
EHR: electronic health record
FHIR: Fast Healthcare Interoperability Resources
HL7: Health Level Seven
ISO: International Organization for Standardization

## References

[1] Lee CH, Yoon HJ (2017). Medical big data: promise and challenges. Kidney Res Clin Pract.

[2] Adel E, El-Sappagh S, Barakat S, Elmogy M, Dey N, Ashour AS, Fong SJ, Borra S (2019). Chapter 13 - Ontology-based electronic health record semantic interoperability: A survey. U-Healthcare Monitoring Systems Volume 1: Design and Applications.

[3] Introduction to HL7 standards. Health Level Seven International.

[4] Standards by ISO/TC 215: Health informatics. International Organization for Standardization.

[5] Clinical models program. openEHR Foundation.

[6] Vida M, Lupse O, Stoicu-Tivadar L (2012). Improving the interoperability of healthcare information systems through HL7 CDA and CCD standards.

[7] Porrasmaa J, Mykkänen J, Tarhonen T, Jalonen M, Kemppainen P, Ensio A, Pakarinen V (2008). Application of HL7 CDA R2 and V3 messaging for national ePrescription in Finland.

[8] Benson T, Grieve G (2016). Principles of Health Interoperability: SNOMED CT, HL7 and FHIR, Fourth Edition.

[9] FHIR overview. HL7 FHIR.

[10] Saripalle R, Runyan C, Russell M (2019). Using HL7 FHIR to achieve interoperability in patient health record. J Biomed Inform.

[11] Leroux H, Metke-Jimenez A, Lawley MJ (2017). Towards achieving semantic interoperability of clinical study data with FHIR. J Biomed Semantics.

[12] Gruendner J, Wolf N, Tögel L, Haller F, Prokosch H, Christoph J (2020). Integrating genomics and clinical data for statistical analysis by using GEnome MINIng (GEMINI) and Fast Healthcare Interoperability Resources (FHIR): System design and implementation. J Med Internet Res.

[13] Mandel JC, Kreda DA, Mandl KD, Kohane IS, Ramoni RB (2016). SMART on FHIR: a standards-based, interoperable apps platform for electronic health records. J Am Med Inform Assoc.

[14] (2019). ISO 13606-1:2019 - Health informatics — Electronic health record communication — Part 1: Reference model. International Organization for Standardization.

[15] Pahl C, Zare M, Nilashi M, de Faria Borges MA, Weingaertner D, Detschew V, Supriyanto E, Ibrahim O (2015). Role of OpenEHR as an open source solution for the regional modelling of patient data in obstetrics. J Biomed Inform.

[16] What is openEHR?. openEHR Foundation.

[17] Min L, Tian Q, Lu X, Duan H (2018). Modeling EHR with the openEHR approach: an exploratory study in China. BMC Med Inform Decis Mak.

[18] Wei P, Atalag K, Day K (2019). An openEHR approach to detailed clinical model development: Tobacco smoking summary archetype as a case study. Appl Clin Inform.

[19] Leslie H (2020). OpenEHR archetype use and reuse within multilingual clinical data sets: Case study. J Med Internet Res.

[20] Li M, Leslie H, Qi B, Nan S, Feng H, Cai H, Lu X, Duan H (2020). Development of an openEHR template for COVID-19 based on clinical guidelines. J Med Internet Res.

[21] Mascia C, Uva P, Leo S, Zanetti G (2018). OpenEHR modeling for genomics in clinical practice. Int J Med Inform.

[22] Moreno-Conde A, Moner D, da Cruz WD, Santos MR, Maldonado JA, Robles M, Kalra D (2015). Clinical information modeling processes for semantic interoperability of electronic health records: systematic review and inductive analysis. J Am Med Inform Assoc.

[23] Moner D, Maldonado JA, Robles M (2018). Archetype modeling methodology. J Biomed Inform.

[24] Helou SE, Kobayashi S, Yamamoto G, Kume N, Kondoh E, Hiragi S, Okamoto K, Tamura H, Kuroda T (2019). Graph databases for openEHR clinical repositories. International Journal of Computational Science and Engineering.

[25] Kalogiannis S, Deltouzos K, Zacharaki EI, Vasilakis A, Moustakas K, Ellul J, Megalooikonomou V (2019). Integrating an openEHR-based personalized virtual model for the ageing population within HBase. BMC Med Inform Decis Mak.

[26] Wang L, Min L, Wang R, Lu X, Duan H (2015). Archetype relational mapping - a practical openEHR persistence solution. BMC Med Inform Decis Mak.

[27] Tian Q, Han Z, An J, Lu X, Duan H (2019). Representing rules for clinical data quality assessment based on openEHR guideline definition language. Stud Health Technol Inform.

[28] Costa CM, Menárguez-Tortosa M, Fernández-Breis JT (2011). Clinical data interoperability based on archetype transformation. J Biomed Inform.

[29] Yang L, Huang X, Li J (2019). Discovering clinical information models online to promote interoperability of electronic health records: A feasibility study of openEHR. J Med Internet Res.

[30] Christensen B, Ellingsen G (2016). Evaluating model-driven development for large-scale EHRs through the openEHR approach. Int J Med Inform.

[31] OpenEHR deployed solutions. openEHR Foundation.

[32] Hak F, Oliveira D, Abreu N, Leuschner P, Abelha A, Santos M (2020). An openEHR adoption in a Portuguese healthcare facility. Procedia Comput Sci.

[33] Pellison FC, Rijo RPCL, Lima VC, Crepaldi NY, Bernardi FA, Galliez RM, Kritski A, Abhishek K, Alves D (2020). Data integration in the Brazilian public health system for tuberculosis: Use of the semantic web to establish interoperability. JMIR Med Inform.

[34] Wulff A, Haarbrandt B, Tute E, Marschollek M, Beerbaum P, Jack T (2018). An interoperable clinical decision-support system for early detection of SIRS in pediatric intensive care using openEHR. Artif Intell Med.

[35] Min L, Tian Q, Lu X, An J, Duan H (2018). An openEHR based approach to improve the semantic interoperability of clinical data registry. BMC Med Inform Decis Mak.

[36] Atalag K, Farris A, Kerr A (2015). Information modelling of ANZACS-QI datasets to support data management using openEHR.

[37] Oliveira D, Miranda FMM, Coimbra A, Abreu N, Leuschner P, Abelha AC (2020). OpenEHR meets interoperability and knowledge engineering. International Journal of Reliable and Quality E-Healthcare.

[38] Clinical knowledge manager. openEHR Foundation.

[39] Leslie H Archetype design patterns. openEHR wiki.

